# Gynotoxic Effects of Chemotherapy and Potential Protective Mechanisms

**DOI:** 10.3390/cancers16122288

**Published:** 2024-06-20

**Authors:** Anna Markowska, Michał Antoszczak, Janina Markowska, Adam Huczyński

**Affiliations:** 1 Department of Perinatology and Women’s Health, Poznań University of Medical Sciences, 60-535 Poznań, Poland; 2 Department of Medical Chemistry, Faculty of Chemistry, Adam Mickiewicz University, 61-614 Poznań, Poland; 3 Gynecological Oncology Center, Poznańska 58A, 60-850 Poznań, Poland; jmarkmed@poczta.onet.pl

**Keywords:** ovarian reserve, ovarian function, ovotoxicity, chemotherapy, protective factors, breast cancer, gynecological cancers

## Abstract

**Simple Summary:**

Though chemotherapy is generally known to be effective in the fight against cancer, its application is associated with a number of side effects, including toxic impacts on the ovaries. The most ovotoxic cytostatic chemotherapeutics are the classical alkylating compounds, particularly cyclophosphamide. Thus, it is of utmost importance to find effective means to protect ovaries against the negative influence of chemotherapeutic agents. This review paper presents the results of the current research work on the hitherto proposed agents potentially protecting the functions and state of ovaries exposed to chemotherapy. A large body of promising results have been reported, but as presented, it is necessary to undertake thorough and comprehensive studies aimed at providing an explicit evaluation of the efficacy of selected ovoprotecting agents and their possible clinical use in the future.

**Abstract:**

Chemotherapy is one of the leading cancer treatments. Unfortunately, its use can contribute to several side effects, including gynotoxic effects in women. Ovarian reserve suppression and estrogen deficiency result in reduced quality of life for cancer patients and are frequently the cause of infertility and early menopause. Classic alkylating cytostatics are among the most toxic chemotherapeutics in this regard. They cause DNA damage in ovarian follicles and the cells they contain, and they can also induce oxidative stress or affect numerous signaling pathways. In vitro tests, animal models, and a few studies among women have investigated the effects of various agents on the protection of the ovarian reserve during classic chemotherapy. In this review article, we focused on the possible beneficial effects of selected hormones (anti-Müllerian hormone, ghrelin, luteinizing hormone, melatonin), agents affecting the activity of apoptotic pathways and modulating gene expression (C1P, S1P, microRNA), and several natural (quercetin, rapamycin, resveratrol) and synthetic compounds (bortezomib, dexrazoxane, goserelin, gonadoliberin analogs, imatinib, metformin, tamoxifen) in preventing gynotoxic effects induced by commonly used cytostatics. The presented line of research appears to provide a promising strategy for protecting and/or improving the ovarian reserve in the studied group of cancer patients. However, well-designed clinical trials are needed to unequivocally assess the effects of these agents on improving hormonal function and fertility in women treated with ovotoxic anticancer drugs.

## 1. Introduction

**Figure 1:**
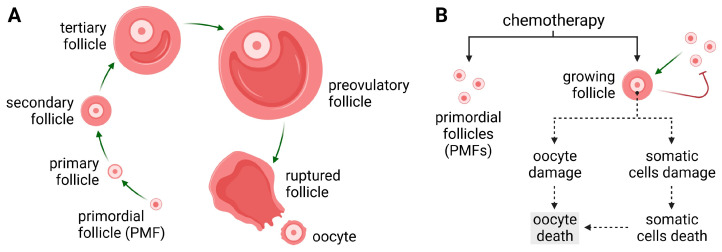
(**A**) Ovarian follicle maturation and (**B**) adverse effects of chemotherapy on the ovarian reserve. Oncological drugs can directly damage PMFs while also reducing the number of growing follicles, thus promoting the maturation of PMFs, which can indirectly affect the reduction of their reserve. Chemotherapeutics can damage oocytes or follicular somatic cells, which leads to oocyte death in both cases. The figure was created with BioRender.com, accessed on 12 April 2024.

The ovary is one of the most important components of the reproductive system. Folliculogenesis, the process of forming the basic ovarian structures—follicles [1]—begins with primordial follicles (PMFs) [2], whose number is determined at birth. They contain a prophase oocyte surrounded by a layer of granulosa cells, which, after activation, mainly by the PI3K/AKT pathway, grows and changes into a primary follicle, then into a secondary follicle, and finally into a pre-ovulatory antral follicle (Figure 1A) [3]. The pool of PMFs does not change substantially throughout a woman’s reproductive life [2], while their loss from the resting pool occurs continuously. Depletion of the PMF pool leads to primary ovarian failure (POF) [4], and a PMF count of less than 1000 can result in menopause [5].

Chemotherapy can have an irreversible impact on women’s fertility, especially in childhood cancers and those most commonly affecting women of reproductive age, such as breast cancer [6]. Despite advances in the development of new cancer treatments, a significant percentage of women experience ovarian dysfunction as a result of chemotherapy (Figure 1B) [7,8,9]. Estrogen deficiency leads to a reduced quality of life for women, including menstrual disorders and menopausal symptoms (night sweats and hot flashes) [8]. It can ultimately lead to fertility disorders or accelerate early menopause. It is estimated that the use of alkylating cytostatics causes ovarian failure among ~40% of women [4,10]. According to other data, depending on a woman’s age, the risk of amenorrhea in cancer patients ranges from 20% to as high as 80% [11]. Other consequences include an increased risk of cardiovascular, skeletal, or neurological symptoms (cognitive impairment) [8].

The gynotoxic effects of chemotherapy cause endocrine dysfunction in women of reproductive age, i.e., 15–39 years [12,13,14]. For this reason, it is recommended that the ovarian reserve should be assessed before treatment with chemotherapeutic agents. These tests should include determination of levels of four hormones—estradiol, inhibin B, follicle-stimulating hormone (FSH), and anti-Müllerian hormone (AMH)—as well as estimation of the number of antral follicles using transvaginal ultrasound examination [15,16]. The AMH levels prior to chemotherapeutic treatment can be an essential factor in determining ovarian function in patients with early-stage breast cancer after completion of therapy [17,18]. The deleterious effects of chemotherapeutics concern both follicles and the ovarian cells within them (Figure 1B), but they also affect signaling pathways related to hormonal functions. Nevertheless, the primary mechanism responsible for the depletion of the ovarian reserve is still ambiguous for some chemotherapeutic agents [19].

The risk of ovarian dysfunction resulting from chemotherapy may depend on the patient’s age or baseline fertility status [8]. Acute ovarian failure is much more common among older women during or immediately after therapy [20,21]. This difference may be due to a much smaller reserve of PMFs at the onset of treatment in older women [22], so it is more likely that the loss of an additional pool of follicles will induce POF at the end of treatment in this group of patients [23]. Nevertheless, it should not be forgotten that chemotherapeutic-induced follicle damage may occur at virtually any age.

According to Nguyen et al. [24], there may be some plasticity in the ovarian reserve that allows fertility to be preserved even in the event of significant loss of PMFs, which may be related to the localization of PMFs in the periphery of the ovary, where cytostatic agents reach to a lesser extent. According to the authors, however, this issue requires further in-depth studies. On the other hand, Zhang et al. [25] showed that primary oocytes are more susceptible to cytostat-induced apoptosis than those entering the growth phase. A study by Xiong et al. [26] described three main pathways of harmful effects of cytostatics on ovarian structures, which are (i) DNA damage mediated by initiating the proapoptotic proteins NOXA and PUMA, (ii) immune effects via the pro-inflammatory interleukins IL-1/6 and the proapoptotic TNF*α*, and (iii) induction of oxidative stress, primarily due to the accumulation of reactive oxygen species (ROS). It should be emphasized that the mechanism and potential for inducing toxic effects on the ovaries depends on the type of cytostatic drug used, its dose, and the therapy regimen [8]. Byrne et al. [27] observed that the risk of early menopause in young women (under 20 years of age) undergoing chemotherapy is up to nine times higher than that among patients aged 21–25 years in the control group. Numerous reports have indicated the possible ovotoxic properties of selected alkylating drugs in this context [28].

## 2. Gynotoxic Properties of Anticancer Drugs

**Figure 2:**
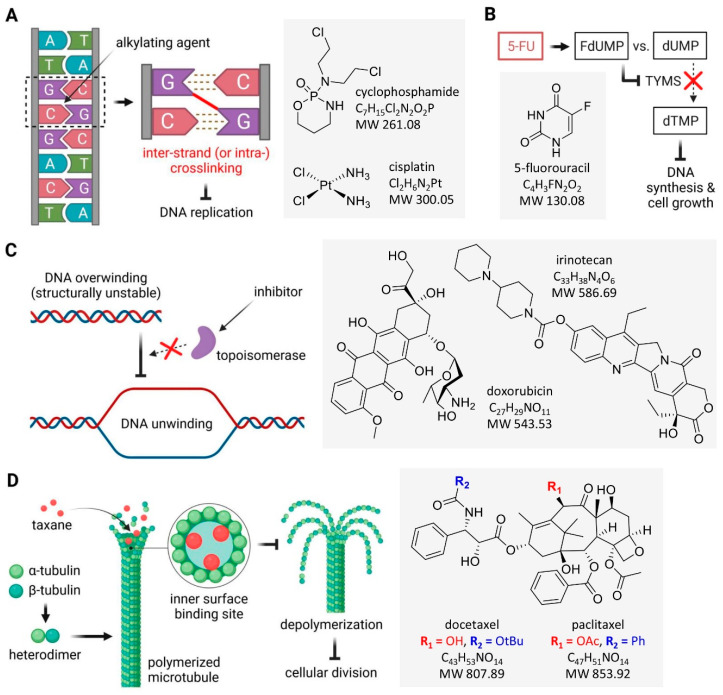
Mechanism of action and structure of selected anticancer drugs with gynotoxic properties from the groups of (**A**) alkylating cytostatics, (**B**) antimetabolites, (**C**) topoisomerase I/II inhibitors, and (**D**) mitosis inhibitors. 5-FU, 5-fluorouracil; FdUMP, 5-fluorodeoxyuridine monophosphate; dUMP, deoxyuridine monophosphate; dTMP, deoxythymidine monophosphate; TYMS, thymidylate synthase. The figure was created with BioRender.com, accessed on 12 April 2024.

The primary criterion of cytostatic division is the anticancer action mechanism of specific anticancer drugs. Among the best-known and most widely used cytostatic drugs are alkylating agents, antimetabolites, topoisomerase I/II inhibitors, and mitosis inhibitors (Figure 2). Significantly, the gynotoxic properties of cytostatic drugs vary not only within these groups but also among individual drugs belonging to the same group. For example, it has been shown that the therapy with classic alkylating drugs, particularly cyclophosphamide, is associated with a significantly higher risk of ovarian failure than with platinum derivatives or anticancer plant alkaloids [10]. The chemotherapy-induced amenorrhea has been assessed to occur in up to ~70% of women with breast cancer treated with cyclophosphamide, 5-fluorouracil, or methotrexate [29]. Lower et al. [30] noted that among premenopausal patients with early-stage breast cancer treated with methotrexate or anthracycline antibiotics, the risk of menstrual abnormalities and menopause increases with age. However, persistent abnormal menstruation occurred in 28% of patients younger than 35, and 30% of all patients did not menstruate for 12 months after the end of chemotherapy [30].

The risk of losing PMFs and experiencing early menopause also rises when increasing the dose of the chemotherapeutic drug used [31,32]. The material obtained using biopsies from cancer patients treated with chemotherapy contains significantly fewer PMFs than in untreated controls. In addition to PMFs, more mature subpopulations of follicles are also susceptible to damage caused by commonly used chemotherapeutic agents [33,34]. The following subsections summarize the gynotoxic properties of selected compounds belonging to the four main groups of chemotherapeutic agents determined in studies performed on either animal models or human ovarian tissues, as listed in Table 1 and Table 2. Moreover, in some cases, the relevant data were available from the studies conducted on oncologic patients. We searched databases, i.e., GoogleScholar and PubMed, for original reports on the treatment of the harmful effects of anticancer drugs on the ovaries that were published mainly in the last 20 years. When searching the databases, we took into account the chemotherapeutics whose potential gynotoxic properties have been most extensively described in the scientific literature. As keywords, we used a combination of the name of the anticancer drug of choice, i.e., “cyclophosphamide”, “cisplatin”, “5-fluorouracil”, “doxorubicin”, “irinotecan”, “docetaxel”, or “paclitaxel”, as well as phrases that may indicate their harmful effects on the ovaries, i.e., “ovotoxicity”, “ovarian failure”, “ovarian reserve”, or “human ovary tissue”. In addition, the literature lists of key articles were checked for relevant papers to cite in this review paper.

### 2.1. Alkylating Drugs

Alkylating drugs are phase non-specific, causing alkylation of DNA and altering its structure by forming adducts through the attachment of an alkyl group to the N^7^ purine ring of the DNA guanine base (Figure 2A). Consequently, it leads to the inhibition of cell division and, ultimately, to cell death. Classic alkylating drugs are among the most ovotoxic anticancer agents [23,29,30,80]. Drugs from this group are used against many types of cancer, but it has been shown that they can cause a loss of ovarian reserve in a dose-dependent manner [32,51,81], which may lead to POF and reduced fertility among cancer patients [82,83]. A study by Brayboy et al. [84] proved that increasing the dose of nitrogen mustard leads to increased oxidative stress in parental oocytes and those of the F1 generation. MDR-1, also known as ABCB1 or P-glycoprotein (P-gp), can protect oocyte mitochondria from the transgenerational effects of nitrogen mustard exposure [85]. Shai et al. [86], in a cohort study on a group of 96 women aged 15–39 who underwent cryopreservation of ovarian tissue, including 48 women with a history of oncology, pointed to a pathological mechanism of action of alkylating drugs contributing to follicle loss.

**Figure 3:**
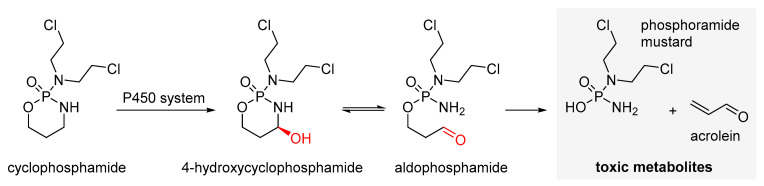
Cyclophosphamide activation pathway.

Cyclophosphamide (Figure 2A) is the most widely used alkylating cytostatic. It is a prodrug metabolized by the enzymes of the cytochrome P450 system in the liver to 4-hydroxycyclophosphamide and aldophosphamide, which are precursors of phosphoramide mustard and acrolein (Figure 3). Phosphoramide mustard is the main active metabolite of cyclophosphamide [87,88], which induces DNA cross-linking, leading to the formation of adducts that prevent DNA replication. Phosphoramide mustard also affects mitochondria, reducing transmembrane potential and leading to the accumulation of cytosolic cytochrome c. Various metabolites of cyclophosphamide also show a range of ovotoxic properties [89]. Cyclophosphamide is widely used against many types of cancer, including childhood cancers, melanoma, and breast cancer, including among oncologic patients who are pregnant [90].

Platinum-based anticancer drugs, despite not having alkyl groups, can bind permanently to DNA, disrupt its repair, and lead to cell death. For this reason, these compounds are classified as “alkylating-like” anticancer drugs. The most important representative of this group of drugs is cisplatin (Figure 2A), which damages the DNA of cancer cells by forming adducts, binds to many nuclear and cytoplasmic proteins, and interferes with numerous molecular pathways. Cisplatin is used as first-line chemotherapy in many malignancies, such as leukemia, lymphomas, sarcomas, and testicular, head and neck, cervical, breast, and ovarian cancer [91]. Unlike standard alkylating drugs, the gynotoxic effects of platinum derivatives are considered moderately harmful. Nevertheless, in women of childbearing age treated for cancer, cisplatin can lead to impaired ovarian function, POF, and infertility [92].

**Figure 4:**
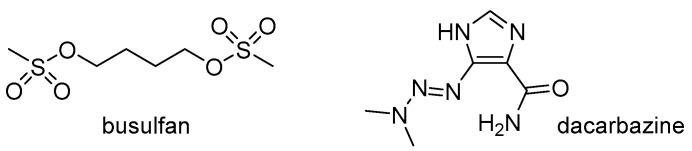
Structure of busulfan and dacarbazine—examples of other alkylating cytostatic drugs with potential ovotoxic properties.

Although the potentially harmful effects of cyclophosphamide and cisplatin on the ovaries have been most extensively described in the scientific literature, it should be kept in mind that other drugs in the alkylating cytostatic group, including busulfan and dacarbazine (Figure 4), may also have significant ovotoxic properties [93,94,95].

#### 2.1.1. Non-Human Studies

In vitro and animal model studies have shown that cyclophosphamide can induce apoptosis in granular layer cells and, in addition, cause inflammation and damage to the blood vessel system, decrease the production of antioxidant enzymes, and induce mitochondrial dysfunction [7,9,96,97,98,99,100,101,102]. On the other hand, the role of apoptosis in PMFs is ambiguous. The results of some studies in a mouse model have provided evidence for apoptosis of follicles, including PMFs, as a result of treatment with cyclophosphamide [43,103,104,105,106], while other reports have not confirmed this [102,107,108,109]. In an in vitro study, phosphoramide mustard (Figure 3) used at a concentration of 10 µM did not induce apoptosis in 90% of precursor cells in the ovary—pre-pubertal and pre-germ cells—even though they underwent extensive DNA damage, cell cycle arrest, and premature aging [110]. According to Lande et al. [111], cyclophosphamide metabolites in vitro appeared to enhance the activation of PMFs toward more mature follicles. Again, no apoptosis was observed in PMFs [111].

The deleterious effects of cyclophosphamide may also result from damage to the mitochondrial membrane of oocytes [68] or the induction of ROS production [36], while inhibition of ovarian granulosa cell proliferation and POF may result from the activation of the lncRNA-Meg3-p53-p66Shc pathway [112]. Blocking the cholesterol biosynthesis pathway with cyclophosphamide has also been identified as a potential inducer of POF [42].

The effect of cyclophosphamide use on the developing ovaries of fetuses was tested in studies conducted on pregnant rodents with cancer; the formation of fewer follicles and accelerated activation of PMFs were found [38,39]. Salian et al. [40] investigated a possible effect of the exposure of female mice to cyclophosphamide on ovarian function and oocyte condition. The authors of this study concluded that exposure to chemotherapy, especially at a young age, can contribute to impaired long-term fertility despite the continued presence of follicles in the ovary [40]. According to Di Emidio et al. [41], cyclophosphamide can adversely affect the competence of the oocytes of the offspring and, thus, reduce the fertility of subsequent generations.

In studies on rat ovarian granulosa cells, it has been shown that cyclophosphamide can reduce the transmembrane potential of mitochondria and cause cytochrome c accumulation in the cytosol, resulting in caspase activation and programmed cell death [37]. On the other hand, reports describing glutathione deficiency as a factor by which cyclophosphamide could induce follicle apoptosis are quite inconclusive [113,114]. Phosphoramide mustard (Figure 3) induced the expression of H2AX protein—a recognized marker of DNA double-strand breaks (DSBs)—in both mouse ovarian granulosa cells and oocytes in in vitro assays [115], and at concentrations of ≥3 µM, it led to a significant loss of PMFs (>90%) and morphological changes, especially in oocytes [89]. Another study in mice indicated that the administration of fairly high single doses of cyclophosphamide (100 mg/kg) on the 21st day after birth led to decreases in the number of remaining ovarian follicles, impaired hemostasis of oocyte quality, and reduced the chance of embryo development [35]. The adverse effects of cyclophosphamide on ovarian and oocyte function persisted after the completion of chemotherapy [44].

Most of the information on cisplatin’s toxicity to the ovaries comes from animal studies. In vitro cultured ovaries of mice treated with cisplatin were characterized by a reduced number of follicles (*p* = 0.001) and an increased number of unhealthy follicles (73%, *p* = 0.001) [71]. Kim et al. [69] noted that both follicle survival and growth were inhibited after cisplatin treatment, with granulosa cells proving more sensitive to the drug than oocytes. On the other hand, mouse studies have shown that cisplatin can cause oocyte death in PMFs from both short-term treatment with high doses of the drug (5 mg/kg) and prolonged treatment with low doses (2 mg/kg once per day for 15 days) [47]. Direct oocyte damage in PMFs by cisplatin has also been noted by other research groups [43,70].

In addition, it has been shown that cisplatin could induce cell death in immature oocytes, which was presumed to be mediated by a non-receptor tyrosine kinase (c-Abl) [116], which, acting as a sensor of DNA damage, could, when activated, affect TAp63-*α*, a p53 homolog expressed in oocytes. The TAp63 pathway has been indicated as an effective target for developing interesting ovarian protective strategies [9,117,118]. Injection of cisplatin into the ovaries of newborn mice resulted in the accumulation of c-Abl and TAp63-*α* in the oocyte, leading to cell death, while the pharmacological inhibition of c-Abl activity resulted in a reduction in the extent of oocyte damage by the drug [119]. Cisplatin may also cause endoplasmic reticulum stress [120] or contribute to ovarian damage through activation of the kallikrein-kinin system (KKS) in response to inflammation and oxidative damage caused by its use [48]. The involvement of the PTEN/AKT/FOXO3 pathway in POF after cisplatin treatment has also been considered [121].

Mitochondrial dysfunction in oocytes induced by cisplatin may lead to the depletion of the ovarian reserve. Nevertheless, the long-term effect of this drug on mitochondria, according to Wang and Hutt [49], may be minimal. The study’s authors noted that immature oocytes that survived cisplatin treatment were able to develop into mature oocytes with normal mitochondrial parameters [49]. Among the other side effects of cisplatin use were decreased estradiol levels and increased gonadotropin levels [50].

#### 2.1.2. Human Tissue Studies

Although cyclophosphamide was the first chemotherapeutic drug to be linked to ovarian dysfunction, amenorrhea, and POF, relatively few papers in the scientific literature have been concerned with the evaluation of the effects of cyclophosphamide on human ovarian cells or tissue. In a study performed on human ovarian cortex sections obtained from premenopausal women and incubated with a cyclophosphamide-containing medium, Raz et al. [34] noted that the cytostatic may have a destructive effect on human follicles, which could, in turn, result from damage to granulosa cells and the basement membrane. However, it should be mentioned that the drug dose causing damage to granulosa cell nuclei (0.5 mg/mL) was higher than the recognized pharmacological level [34]. On the other hand, however, cancer patients are often given high doses of cyclophosphamide continuously for up to 4 days, resulting in high levels of circulating cyclophosphamide [34]. In a study on a human ovarian xenograft model in mice, it was noted that follicle loss at 12 h, 24 h, and 48 h after cyclophosphamide administration was 12%, 53%, and 93%, respectively [46].

Based on the available literature, it is difficult to determine the specific effects of cyclophosphamide on PMFs. While some studies indicate an indirect effect of cyclophosphamide in reducing the population of PMFs [122], other reports report direct damage to this population of follicles by the drug [45]. In studies on human granulosa cells, cyclophosphamide has been shown to inhibit ovarian development by increasing levels of N^6^-methyladenosine, which is associated with the expression levels of methyltransferases, demethylases, and RNA effectors [123]. Apoptosis in human COV434 granulosa cells included oxidative stress and glutathione deficiency [114]. The effect of the active metabolite cyclophosphamide on growing follicles in human ovarian tissue was also noted in vitro, where increased apoptosis of granulosa cells and atresia (overgrowth) of follicles was observed [72,124].

In contrast, the use of cisplatin led to apoptosis of human ovarian stromal tissue and reduced cell proliferation [73]. In studies on human ovarian cortex fragments and granulosa cells, treatment with cisplatin resulted in reduced follicle number and steroidogenic activity [72,125].

#### 2.1.3. Effects on Cancer Patients

Patients treated with cisplatin usually receive it in combination with other anticancer agents, making it difficult to determine the direct toxic effects of the drug on the ovaries. Maneschi et al. [126] described the risk of amenorrhea after treatment with a multidrug regimen, including cisplatin, as mild to moderate and the risk of ovarian failure and infertility as high. Other authors have noted no deleterious effects of cisplatin-based monotherapy on fertility, especially for germ cell tumors [127,128].

### 2.2. Antimetabolites

The action of cytostatics belonging to antimetabolites may involve the incorporation of false building blocks into the DNA structure or blocking the incorporation of molecules necessary for normal DNA synthesis. Mechanistically, the anticancer action of 5-fluorouracil (Figure 2B) includes the incorporation of active metabolites into the structure of nucleic acids [129] or the conversion of this drug into 5-fluorodeoxyuridine monophosphate (FdUMP), which then binds to thymidylate synthase (TYMS), inhibiting the production of deoxythymidine monophosphate (dTMP), which is essential for DNA replication and repair (Figure 2B) [130,131]. In addition, 5-fluorouracil plays an important role in the fight against colon, head and neck, and breast cancers [132]. Although antimetabolic drugs are not considered particularly harmful to the ovaries, it has not been clearly defined what risks are related to the use of multiple doses of this type of cytostatic on long-term fertility in women. However, there are a growing number of reports in the scientific literature on the potential ovotoxic properties of 5-fluorouracil, which are described in the following subsections.

#### 2.2.1. Non-Human Studies

Female mice in the estrous phase were more susceptible to the negative effects of 5-fluorouracil, i.e., increased risk of loss of fertility, than those in the metestrus, diestrus, or proestrus phases [133,134]. In order to reduce reproductive impairment, it, therefore, seems advisable to evaluate and select the appropriate estrous cycle before using 5-fluorouracil therapy.

Exposure of adult female mice to 5-fluorouracil did not result in changes in the number of PMFs and primary follicles, although atresia of secondary and antral follicles increased significantly, and the number of corpus luteum decreased after administration of this cytostatic, leading to a decrease in ovarian volume [52,54]. After a week, however, the frequency of atresia returned to a level similar to that in the control group [52], suggesting that reproductive activity after the use of 5-fluorouracil can be restored by continued follicular growth. Similar results have been presented by other authors—repeated intraperitoneal administration of 5-fluorouracil to adult female mice resulted in a reduction in ovarian size and the number of corpus luteum, consequently leading to ovulatory dysfunction [55]. However, these disadvantages could be reversed, and no apparent abnormalities were observed in the offspring [55].

Almeida et al. [53], on the other hand, noted that injection of 5-fluorouracil into young female mice causes a complete loss of secondary follicles. Moreover, the genes involved in apoptosis and the Wnt signaling pathway were expressed when the ovaries of young mice were cultured in vitro with 5-fluorouracil [53]. In a study of rats treated with 5-fluorouracil at a dose of 100 mg/kg, significantly higher vascular congestion, edema, follicular degeneration, and leukocyte infiltration were observed compared to those in the control group [135]. In another study on rats treated with 5-fluorouracil, increased levels of malondialdehyde (MDA), total oxidant status (TOS), and the oxidative stress index (OSI) were observed in the ovaries compared to the control group (*p* = 0.011, *p* = 0.003, and *p* = 0.001, respectively) [136].

In vitro studies on mouse secondary follicles revealed that 5-fluorouracil does not affect follicle morphology, but depending on the concentration used, this drug can significantly reduce follicle size [74].

#### 2.2.2. Human Tissue Studies

As the determination of the developmental and reproductive toxicity induced by 5-fluorouracil directly in humans is highly problematic, studies using human-induced pluripotent stem cells (hiPSCs) are useful in this context [137]. In studies on hiPSCs, 5-fluorouracil was found to inhibit neural differentiation by reducing the expression of mitochondrial Mfn1/2 fusion proteins and intracellular ATP levels [138], identifying mitochondrial dysfunction as a possible source of 5-fluorouracil’s potential gynotoxic properties.

#### 2.2.3. Effects on Cancer Patients

The use of 5-fluorouracil or its combination with leucovorin and oxaliplatin (FOLFOX regimen) in the treatment of women in the second/third trimester of pregnancy had no harmful effects on fetal health [139,140]. The reproductive toxicity of 5-fluorouracil is presumed to be reversible, and some drugs used in combination with 5-fluorouracil during chemotherapy may protect the reproductive system of both women and men from its potentially harmful effects [96].

### 2.3. Topoisomerase Inhibitors

**Figure 5:**
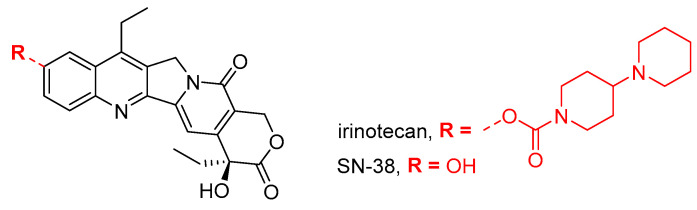
Structural differences between irinotecan and the active metabolite SN-38.

By binding to DNA, topoisomerases I and II enable it to relax during replication (Figure 2C). A number of inhibitors of these enzymes are known, of which doxorubicin (an anthracycline antibiotic as a topoisomerase II inhibitor) and irinotecan (the active metabolite of SN-38 as a topoisomerase I inhibitor) (Figure 5) have found use as anticancer drugs. It should be noted that doxorubicin may cause DNA damage not only through a pathway involving topoisomerase II but also through intercalation into DNA or through oxidative stress.

Doxorubicin and irinotecan are active against a variety of tumor types, including lung, gastric, ovarian, breast, or hematologic cancers [141,142]. Cardiotoxicity occurs with all currently clinically available anthracyclines. On the other hand, a growing body of literature, especially that concerned with doxorubicin, indicates that among the possible side effects of topoisomerase inhibitors, their potential gynotoxicity should also be considered. According to some studies, the likelihood of missing menstruation after doxorubicin treatment can range from 7% to as high as 80%, depending on the woman’s age and the duration of exposure to the drug [20,58,143].

#### 2.3.1. Non-Human Studies

A single injection of doxorubicin (7.5 mg/kg or 10 mg/kg) into mice resulted in a reduction in both ovarian size and weight, which persisted for one month after treatment [58]. One week after doxorubicin treatment, a reduction in ovulation rate was observed, with partial improvement in this condition after one month [58]. Other research teams have also documented the direct effect of doxorubicin on the ovaries [57,144,145,146].

In a more detailed study, doxorubicin was found to accumulate in the medullary stromal cells of the ovary and then redistribute outward to the cortex and follicles in a time-dependent manner [147]. Zhang et al. [75] demonstrated that doxorubicin, in a dose-dependent manner, may decrease mitochondrial membrane potential, increase ROS levels, and induce apoptosis in granulosa cells isolated from mouse ovaries. In addition to inducing granulosa cell death in growing follicles, a possible role for doxorubicin in reducing proliferating cell nuclear antigen-positive endothelial cells in the corpus luteum and interfering with F-actin in luteal cells has also been indicated [59]. In parallel, in in vitro and in vivo studies, Bar-Joseph et al. [57] tested the effects of doxorubicin on germ follicle oocytes and showed that the drug is able to penetrate the blood-tubule barrier, accumulating in chromatin in granulosa cells and oocytes. Moreover, germ follicle oocytes have been found to be more susceptible to doxorubicin than the ovulated second meiotic division (MII)-arrested oocytes [57]. Using various animal models, the effects of doxorubicin on all stages of follicle development have also been studied [56]. The authors of this study showed that doxorubicin depletes the ovarian reserve of mice through both atresia of PMFs and their overactivation, while ovotoxicity induced by doxorubicin exposure was age-dependent in the animals [56]. In addition, doxorubicin can lead to the loss of PMFs by affecting the TAp63 pathway [148]. Negative effects of doxorubicin on the condition of rat ovaries and ovarian follicles have also been demonstrated [60,149,150], including those on 3D-cultured follicles [149].

Doxorubicin can disrupt Ca^2+^ homeostasis, which affects transmembrane potential and mitochondrial permeability [151]. In a study conducted on secondary ovarian follicles treated with doxorubicin (200 nM), an increase in Ca^2+^ levels in the cytosol was noted, while Ca^2+^ levels in the endoplasmic reticulum decreased and remained low throughout the study [77], clearly demonstrating that doxorubicin use can cause calcium release from the endoplasmic reticulum. This is important in the context of the drug’s potential harm to the ovaries, since calcium balance is essential for normal oocyte physiology, ovarian follicle growth, and the regulation of gonadotropin secretion. On the other hand, doxorubicin had a negative effect on 17*β*-estradiol (E2) secretion [76].

Importantly, any strategy to reduce the adverse effects of chemotherapeutic agents on ovarian status may require finding the right combination of drugs. For example, imatinib protected the ovaries of newborn mice from damage caused by cisplatin but not that of doxorubicin, even though both anticancer drugs induced ovarian damage, but in markedly different ways [70].

Reports on the potential gynotoxic properties of irinotecan are much less numerous than those of doxorubicin. Levi et al. [61] found in a study on mice that irinotecan causes mild ovotoxicity. Administration of irinotecan (100 mg/kg) induced acute ovarian cell apoptosis, reduced vascularization, and mild, statistically significant, long-term reductions in the number of growing follicles, ovarian weight, and ovarian reserve [61]. By inducing Fas ligand (FasL) expression, irinotecan can induce programmed granulosa cell death of large follicles [62]. The Fas/FasL pathway has been linked to granulosa cell apoptosis during follicle atresia, as well as via the p53 protein [152]. Irinotecan-induced ovarian follicle apoptosis can be attenuated by the deletion of the death-associated protein kinase (DAPK) domain [153]. In contrast, the irinotecan metabolite SN-38 (Figure 5) induced germ cell loss in the testes but not in the ovaries of young mice before puberty [154].

#### 2.3.2. Human Tissue Studies

The use of doxorubicin proved more detrimental to human primary ovarian granulosa cells isolated from follicular fluid aspirates than other chemotherapeutic agents used in the trials (cyclophosphamide, vincristine, methotrexate) [11]. The study’s authors observed a significant reduction in the viability of these cells [11]. An in vitro study using ovarian sections taken from 14 women at the time of cesarean section tested the effect of doxorubicin and its combination with cisplatin on human tissue [73]. Ovarian stromal tissue exposed to doxorubicin was characterized by increased apoptosis and decreased cell proliferation [73]. Importantly, no clear evidence has been obtained to indicate the multiplicative interaction between doxorubicin and cisplatin [73].

#### 2.3.3. Effects on Cancer Patients

The purpose of the analysis by Machet et al. [155] was to determine the effect of ABVD chemotherapy (doxorubicin = adriamycin, bleomycin, vinblastine, dacarbazine) on fertility in women treated for Hodgkin’s lymphoma. In the group of women taking ABVD, there were no clear differences in the number of pregnancies, deliveries, or time needed to become pregnant compared to the control group [155].

On the other hand, a clinical study on a group of 32 gynecologic cancer patients tested the effect of combination chemotherapy with irinotecan on the hypothalamic-pituitary-ovarian endocrine system [156]. It has been observed that the use of irinotecan may cause estrogen-rescued menopausal malaise-like symptoms (MMLS) among peri-menopausal cancer patients or secondary amenorrhea in young women [156]. On the other hand, endocrinological and histopathological studies have shown that irinotecan leads to the loss of ovarian follicles and ovarian failure in a short period of time without affecting the secretion of hypothalamic and pituitary hormones [156]. According to the authors of this study, irinotecan shows severe toxicity to the ovaries, and repeated administration of this drug can lead to loss of ovarian follicles, as well as premature ovarian failure, also in young women [156].

### 2.4. Mitosis Inhibitors

Mitosis inhibitors act by inhibiting microtubule polymerization/depolymerization and belong to the group of phase-specific drugs. Taxanes, binding to the *β*-tubulin subunit, inhibit microtubule depolymerization, stabilize microtubules, interfere with cell division in the G/M phase, prevent cell cycle progression, and induce apoptosis (Figure 2D). Paclitaxel (Figure 2D), first approved for the treatment of advanced ovarian cancer in 1992, was subsequently approved for several other cancers, including metastatic breast cancer [157]. Docetaxel (Figure 2D), the second of the first-generation taxanes, was approved by the Food and Drug Administration (FDA) for oncological practice in 1996 [157]. Limitations to the effective use of taxanes stem from their poor solubility, drug resistance acquired by cancer cells during treatment, and bone marrow suppression when using excessively high drug doses [157,158,159]. The potential gynotoxic properties of these drugs should also be considered. Although the toxicity of paclitaxel to the ovaries may be considered mild and transient [63], the inclusion of paclitaxel in the classic breast cancer treatment regimen (cyclophosphamide, doxorubicin) may increase the risk of ovarian damage after therapy [21]. On the other hand, according to Chaqour et al. [160], the use of taxane-based therapy late in pregnancy may have a significant impact on the long-term reproductive health of children in subsequent generations.

#### 2.4.1. Non-Human Studies

It has been documented that the use of drugs from the taxane family can lead to gonadal toxicity, and it may result in high FSH levels [14]. The effects of paclitaxel given intravenously at the maximum tolerated dose (MTD) on morphology and ovarian function were studied in female rats [65]. Paclitaxel mainly affected PMFs, bilayers, and multilayered follicles, while the number of Graaf follicles and corpus luteum did not decrease [65]. Subsequently, a reduction in ovarian reserve was also noted compared to the control group [65]. Additionally, Yucebilgin et al. [51], in a study on rats, concluded that the number of PMFs decreased after high doses of paclitaxel (7.5 mg/kg), which was consistent with the observations of other authors [161], while the addition of a gonadoliberin agonist to a paclitaxel-based treatment regimen led to the protection of follicles and oocytes [64,162]. Simultaneous administration of paclitaxel and carboplatin caused cumulative ovarian damage and infertility in mice with BRIP1 (BRCA1-interacting protein C-terminal helicase-1) mutations [163]. Maidarti et al. [78] examined the effects of paclitaxel on ovarian follicle development and the maturation process of oocytes mechanically dissected from 14-day-old female mice. The follicle maturation in the groups receiving low and medium doses of the drug was almost half that observed in the control group [78]. The high dose of paclitaxel destroyed follicles before day 12 of culture and resulted in significantly greater shrinkage of ovarian follicles and oocytes compared to both the control group and the groups receiving a low or medium dose of the drug [78]. In contrast, no effect of paclitaxel on the meiotic maturation of oocytes was observed [78]. At concentrations of 10^−9^ M or higher, paclitaxel significantly suppressed growth and negatively affected the condition of ovarian follicles, while additive effects were observed in the group receiving a combination treatment with cisplatin (*p* < 0.01) [69]. Importantly, the oocyte-specific genes growth differentiation factor 9 (GDF9) and bone morphogenetic protein 15 (BMP15) were more suppressed by paclitaxel than by cisplatin [69].

In mouse studies, it has been shown that ovarian weight, the number of secondary follicles, and the total number of follicles decrease after docetaxel administration [66]. In addition, increased expression levels of caspase 3 and the proapoptotic protein Bcl-2 have been reported [66]. Another mouse study also confirmed a reduction in the total number of follicles after docetaxel [67]. In addition, the destruction of ovarian structure and induction of γ-H2AX PMF expression were observed [67]. Docetaxel mainly affects the granulosa cells of early-growing follicles, which appears to be closely related to the high mitotic activity of these cells. Lopes et al. [79], while exposing whole ovaries of newborn mice to clinically relevant doses of docetaxel, showed that the number of PMFs significantly decreased after the administration of 10 µM of the drug compared to the control group. Docetaxel induced a significant increase in the expression of cleaved caspase 3 and caspase 8 in a dose-dependent manner [79]. In addition, an increase in the levels of Bax proteins and cleaved PARP was observed compared to *β*-actin, as well as a decrease in the mitochondrial-to-cytosolic cytochrome c ratio [79], suggesting that the mechanism of docetaxel-induced damage may be the activation of the mitochondrial-dependent apoptosis pathway.

#### 2.4.2. Effects on Cancer Patients

Long et al. [164] evaluated the effects of two different chemotherapy regimens, i.e., DTC (docetaxel + pyrarubicin + ifosfamide) and CAF (tegafur + pyrarubicin + ifosfamide), on the development of POF among 164 women (DTC regimen, n = 75; CAF regimen, n = 89) of reproductive age with breast cancer. The patients were subjected to a given treatment regimen for six months, while the effects of the therapy were observed for more than a year after its termination [164]. The incidence of POF was significantly higher in the DTC group [164]. The percentage of patients with eumenorrhea, dysmenorrhea, or absence of menstruation in this group was significantly different from that observed in the group receiving CAF [164]. Adverse changes in serum FSH, luteinizing hormone, and E2 concentrations were also more severe in the DTC group [164].

## 3. Potential Ovarian-Protective Mechanisms

Protecting the ovaries from damage and fertility disorders associated with exposure to chemotherapeutic agents created a new field, oncofertility, which was concerned with the preservation of ovarian hormonal function and fertility and the avoidance of POF [8,26,96,165]. Several procedures have been developed to preserve fertility in girls and women of reproductive age. Scientific societies, including the American Society for Reproductive Medicine (ASRM), recommend oocyte or ovarian tissue cryopreservation (OTC) as an effective and safe procedure in some cases [26,96,165]. Freezing ovarian fragments with subsequent transplantation and sometimes embryo freezing are also used [96,166,167].

**Figure 6:**
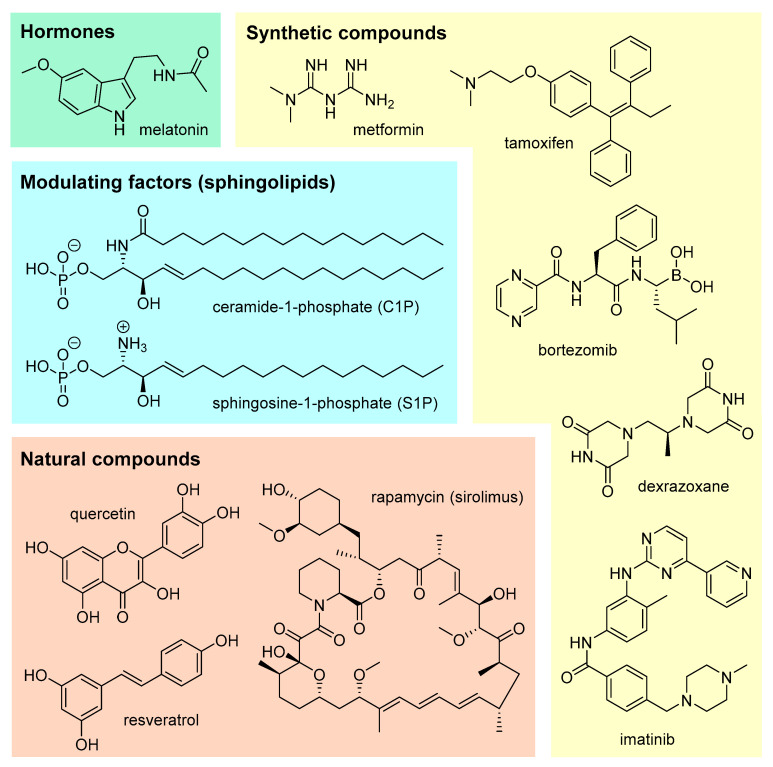
Structure of selected factors with potential ovarian-protective properties during chemotherapy.

In view of the steadily increasing number of patients who are fighting or have won their battle with cancer, as well as the potential gynotoxic properties of commonly used cancer drugs, it seems crucial to search for alternative methods that could protect women’s reproductive functions. In particular, the agents that could protect the ovaries from the toxic effects of chemotherapeutics or alleviate the symptoms of lack of fertility associated with premature menopause are of interest. There are a growing number of reports in the scientific literature on the agents that could protect female fertility and mitigate the adverse effects of chemotherapy, including selected hormones, agents that affect the activity of apoptotic pathways and modulate gene expression, and natural or synthetic chemical compounds (Figure 6).

In order to systematize the state of the art in this area, we searched journal databases, i.e., Google Scholar and PubMed, in detail for original scientific articles on this topic that were published over the last two decades. The keywords were either the name of a potential protective agent, viz. “anti-Müllerian hormone (AMH)”, “ghrelin”, “luteinizing hormone (lutropin)”, “melatonin”, “C1P”, “S1P”, “microRNA (miRNA)”, “quercetin”, “rapamycin”, “resveratrol”, “bortezomib”, “dexrazoxane”, “goserelin”, “leuprorelin”, “leuprolide”, “triptorelin”, “imatinib”, “metformin”, or “tamoxifen”, or a selected drug showing gynotoxic properties, i.e., “cyclophosphamide”, “cisplatin”, “5-fluorouracil”, “doxorubicin”, “irinotecan”, “docetaxel”, or “paclitaxel”. The lists of references in the selected articles were also checked to identify additional literature items. Most studies focused on the potential protective properties of selected agents in studies on animal models (Table 3), and only a few were in vitro studies, including those of human ovarian tissue (Table 4). Studies of the effects of GnRH analogs on cancer patients are more numerous than those of the impact of other groups of potential ovoprotective agents.

### 3.1. Hormones

#### 3.1.1. Anti-Müllerian Hormone

Anti-Müllerian hormone (AMH), also known as Müllerian inhibiting factor (MIF), is produced by preantral and antral follicles in the ovaries. AMH is a recognized biomarker of the ovarian reserve and a negative regulator of PMF activation, and it is produced by the granulosa cells of growing follicles; serum AMH levels of <1.0 ng/mL indicate reduced ovarian reserve [6,9,117,215]. AMH levels drop rapidly during chemotherapy to a much greater extent than estradiol or inhibin B [12,13,14]. Given its low toxicity, high specificity against tumors expressing a specific receptor, and ability to inhibit the proliferation of drug-resistant tumor cells, recombinant AMH appears to be an interesting candidate to support the effects of cancer drugs [216], as well as to limit their deleterious effects on the ovaries [217]. On the other hand, several scientific articles have reported the possible use of AMH as a potential new method to limit the decline of PMFs during chemotherapy [6,168,169,170,207].

A study on adolescent mice treated with simultaneous injections of cyclophosphamide and AMH demonstrated a protective effect of the glycoprotein hormone on ovarian function [168]. The ovaries of cyclophosphamide-treated mice were devoid of PMFs, while in the group exposed to combined administration of cyclophosphamide and AMH, the numbers of both PMFs and early-growing follicles were similar to those observed in the control group [168]. Significantly, 15 weeks after the end of treatment, the number of ovulated eggs after ovarian stimulation was not reduced much in mice treated with cyclophosphamide and AMH [168]. The PI3K signaling pathway is a crucial regulator of many biological processes, including ovarian function, while its dysregulation may contribute to infertility [7]. In this context, mechanistic studies showed that phosphorylation of FOXO3a, a key target of the PI3K/AKT pathway, was lower in the ovaries of mice that were additionally treated with AMH [168]. In the same animal model, Roness et al. [169] confirmed the beneficial effects of AMH on reducing follicle activation and loss of PMFs, as well as improving reproductive outcomes. At the same time, AMH administration did not interfere with the antitumor effects of cyclophosphamide in in vitro tests performed against a breast cancer cell line and in vivo tests on a human leukemia model [169].

According to Man et al. [170], AMH provided a protective effect against cyclophosphamide in both mouse studies and human heterograft models. According to the study’s authors, intraovarian administration of AMH prior to cyclophosphamide may be a relatively non-invasive method of limiting the adverse effects of the chemotherapeutic agent [170]. In tests on ovarian cortex biopsies taken from healthy women, AMH was confirmed to attenuate cyclophosphamide-induced effects, including impaired PMFs and follicle transition health, as well as increased PI3K signaling [207].

The results of a study by Kano et al. [6] conducted on an animal model indicate that the addition of AMH during therapy with cyclophosphamide, carboplatin, or doxorubicin significantly protects the ovarian reserve. In contrast, parenteral administration of AMH with gene therapy arrested follicular development and prevented POF [6].

#### 3.1.2. Ghrelin

Ghrelin is an enteroendocrine peptide, a “hunger hormone” secreted in cells of the gastrointestinal tract, and its ability to mitigate the side effects of chemotherapy, including cachexia in cancer patients [218] or cisplatin-induced testicular toxicity shown in animal studies [219,220], has generated considerable interest in the scientific community. Ghrelin administered in high doses prevented cisplatin-induced ovarian damage by preserving the number of PMFs [171]. A study by Jang et al. [172] conducted on a mouse model proved that ghrelin could help enhance the protective effect of melatonin against cisplatin-induced ovarian failure. The synergistic action of ghrelin and melatonin inhibited PTEN/FOXO3a phosphorylation, which increased the binding affinity of FOXO3a to the p27 protein [172]. This was crucial in “putting to sleep” (dormant status) PMFs, thereby protecting them from apoptosis and damage [172].

#### 3.1.3. Luteinizing Hormone (Lutropin)

Luteinizing hormone (LH), or lutropin, is a glycoprotein hormone secreted by the gonadotropic cells of the anterior lobe of the pituitary gland. An increase in LH levels causes ovulation and the development of corpus luteum in women. The role of gonadotropins, including LH, in the development of ovarian cancer is still ambiguous. While some researchers have found no link between LH levels and epithelial ovarian cancer cell proliferation [221], others have concluded that LH may have either an inhibitory or stimulatory effect [222,223,224,225].

Rossi et al. [173] reported that LH could protect the primary follicle pool during cisplatin administration. Administration of a single dose of LH to young female mice along with cisplatin inhibited the depletion of the PMF reserve and maintained rodent fertility, preventing significant changes in the number of pregnancies and offspring [173]. However, it should be noted that LH may attenuate the antitumor effects of cisplatin and contribute to the development of drug resistance in ovarian cancer cells [226,227].

#### 3.1.4. Melatonin

Melatonin (Figure 6) is a hormone produced by pituitary cells and is responsible for the proper functioning of the so-called “biological clock”. This hormone may have beneficial effects on germ cells during chemotherapy [228]. It may also have a beneficial effect contributing to the prevention of selected cancers or the reduction of the side effects of chemotherapy and/or radiation therapy [229,230,231]. On the other hand, it may improve the quality of life and protect the ovarian function of cancer patients [232].

The main mechanisms by which melatonin may protect ovaries against the damage related to cisplatin-based therapy are the stimulation of the secretion of antioxidant enzymes and the neutralization of free oxygen radicals [7,26]. The results of a study by Xing et al. [174] on mice proved that melatonin may prevent the decrease in the ovarian reserve induced by cisplatin, alleviate disturbances in the cell cycle, inhibit ovaritis, and protect against damage to mitochondria caused by this drug. Other authors have shown that melatonin may not only reduce the toxic effect of cisplatin on the ovaries and protect long-term fertility in mice, but it may also enhance the anticancer activity of this therapeutic agent [175]. Administration of melatonin prevented the depletion of the ovarian reserve in granulosa cells [175]. In female rats, melatonin was found to be able to mitigate dysfunction of the ovaries through changes in steroidogenesis, alleviation of inflammation, and apoptosis, and it reduced oxidative stress and modulated the PTEN/PI3K/AKT/mTOR/AMPK signaling pathway [176]. Melatonin administration attenuated cisplatin-induced follicle loss by preventing the phosphorylation of PTEN/AKT/FOXO3a pathway elements [233]. Barberino et al. [234] documented the protective properties of melatonin against the ovotoxic effects of cisplatin in animal models. However, the effects of melatonin on ovarian function during cisplatin treatment are inconclusive [47], indicating the need for further research in this area.

Melatonin can reduce the adverse effects of not only cisplatin but also cyclophosphamide. For example, Feng et al. [177] demonstrated that melatonin use could prevent PMF activation and litter size reduction among cyclophosphamide-induced mice. Melatonin prevented the loss of ovarian granulosa cells by inhibiting the mitochondrial apoptosis pathway [177]. Melatonin’s interaction with the MT1 receptor and modulation of PTEN/AKT/FOXO3a also attenuated cyclophosphamide-induced loss of PMFs in mice [178]. Pretreatment with melatonin prior to cyclophosphamide administration maintained normal sex hormone levels, improved ovarian follicle morphology and granulosa cell proliferation, and reduced programmed cell death [179].

### 3.2. Modulating Factors

#### 3.2.1. Sphingolipids

Ceramide-1-phosphate (C1P) (Figure 6) is a membrane sphingolipid with potential properties protecting the ovarian function. Administration of C1P reduced cyclophosphamide-induced ovarian damage and protected the ovarian reserve by inhibiting apoptosis and improving vascularization [102]. Sphingosine-1-phosphate (S1P) (Figure 6) is another example of a sphingolipid involved in several physiological processes, including apoptosis of ovarian follicles. The sphingomyelin pathway has been shown to regulate oocyte developmental death, while S1P protects the ovarian reserve from irradiation-induced damage [235]. In an animal model, it was further shown that injection of S1P directly into the ovaries reduces chemotherapy-induced programmed death of PMFs, thereby protecting animal fertility [180,181]. On the other hand, studies on human ovarian xenografts have documented the ability of S1P to block cyclophosphamide- and doxorubicin-induced apoptotic follicle death and preserve the primary follicle supply [45,122]. Of note, S1P reduced the atresia of PMFs during the slow freezing and thawing of human ovarian cortical strips, demonstrating the potential protective role of this sphingolipid [236].

The above-mentioned reports suggest that selected sphingolipids could be an attractive preventive option for the ovarian reserve in women receiving chemotherapy. However, it should be remembered that C1P and S1P must be administered continuously or through injection directly into the ovaries [117].

#### 3.2.2. MicroRNA

MicroRNA (miRNA) is a small non-coding RNA that regulates about 50% of human protein-coding genes. In addition, miRNA can protect PMFs and granulosa cells from atresia and promote the chemosensitivity of cancer cells to anticancer drugs by suppressing molecular DNA repair factors [182,208,237], revealing miRNA as an interesting object of study for a potential strategy to protect the ovarian reserve.

Alexandri et al. [208] demonstrated that miRNA are differentially expressed during exposure to chemotherapeutic agents. Of these, let-7a was the most downregulated, and its delivery prevented the upregulation of genes involved in cell death and reduced chemotherapy-induced apoptosis of mouse ovaries [208]. In contrast, Xiao et al. [182] have shown that the downregulation of two miRNA (miR-10a and miR-146a) in exosomes derived from amniotic fluid stem cells attenuates the anti-apoptotic effect on chemotherapy-damaged granulosa cells. Exosomal miRNA-17-5P derived from human umbilical cord mesenchymal stem cells improved ovarian function by regulating SIRT7 [238].

### 3.3. Natural Compounds

#### 3.3.1. Quercetin

Flavonoids exhibit a broad spectrum of health-promoting properties; unfortunately, the human body cannot biosynthesize such polyphenolic compounds. For this reason, they must be supplied with food. Quercetin (Figure 6) has many natural sources. Nevertheless, it is most abundant in berries, onions, grapes, cherries, broccoli, and citrus fruits [239]. Quercetin is a potent antioxidant flavonoid, and its potential beneficial effects include cardiovascular protection and anti-infective, anti-ulcer, or anticancer effects [240]. Recently, a growing number of studies have also described the possible protective effects of quercetin on reproductive function, including the properties that protect ovarian function due to reduced oxidative stress [241].

Histological analysis by Li et al. [183] showed that quercetin alone did not affect the number of PMFs and the total number of follicles in mouse ovaries compared to the control group. In contrast, the number of early-growing follicles was lower in the group receiving high doses of quercetin (40 mg/kg) [183]. Adding quercetin increased the number of PMFs and the total number of follicles compared to the group receiving cyclophosphamide alone; however, it did not protect early-growing follicles from the effects of the chemotherapeutic agent [183]. Quercetin attenuated cyclophosphamide-induced POF due to reduced mitochondrial oxidative stress and pyroptosis (a type of lytic cell death) in granulosa cells [184].

Results similar to those described above [183] were obtained by Zheng et al. [185] in a study on rats. Again, the numbers of PMFs, primary, secondary, and antral follicles were higher in the group receiving quercetin in combination with cyclophosphamide than in the group receiving the alkylating cytostatic alone [185]. Mechanistically, quercetin inhibited the expression of mRNA, PI3K, AKT, and FOXO3a, suggesting that the compound may restore ovarian function and block oxidative stress as a result of regulation of the PI3K/AKT/FOXO3a signaling pathway [185]. The beneficial protective effects of quercetin on the function of cyclophosphamide-treated ovaries in rat studies have also been confirmed by other authors [186]. A combination of quercetin with rosuvastatin (a hypolipemic drug belonging to the statin group) [242] or capsaicin [243] also produced a positive effect on reducing ovarian damage in animal models.

In turn, Algandaby [187] showed that quercetin could affect the protection of rat ovaries from the toxic effects of another cancer drug, cisplatin. Quercetin exhibited antioxidant and antiapoptotic effects, as evidenced by reduced caspase 3, as well as the modulation of Bax and Bcl-2 expression, and it reduced inflammatory responses in the ovaries [187].

#### 3.3.2. Rapamycin

Rapamycin (sirolimus) (Figure 6) is a macrolide antibiotic that was first isolated in 1975 from the bacterium *Streptomyces hygroscopicus* [244]. Rapamycin is used in transplantation as an immunosuppressive drug, and its potential ovoprotective effects have attracted considerable interest in the scientific community. The use of rapamycin prevented follicle activation in phosphatase and tensin homolog deleted on chromosome ten (Pten)-deficient mice [245], and it maintained oocyte developmental potential in mice and follicle reserves in human cortical fragments implanted into immunodeficient mice [246]. Other reports indicate that rapamycin can protect the ovarian reserve after the cryopreservation or transplantation of ovarian tissue [247].

Zhou et al. [109] demonstrated that the use of rapamycin in combination with cyclophosphamide protects against primary activation and follicle loss caused by the alkylating cytostatic. Other authors have also reported the positive effects of rapamycin on reducing the ovotoxic properties of cyclophosphamide in animal models [188]. In turn, Xie et al. [189] indicated rapamycin’s ability to protect the pool of PMFs during cisplatin treatment in both in vitro and in vivo tests through the compound’s inhibitory effect on the mTOR pathway.

#### 3.3.3. Resveratrol

Resveratrol (Figure 6) is one of the non-flavonoid polyphenols showing antioxidant and anticancer properties [248,249]. The antioxidant properties of resveratrol are associated with the activation of the SIRT1/FOXO1 pathway [7]. The essential element of resveratrol’s structure is a *trans*-stilbene scaffold complemented by three hydroxyl groups at the positions C3, C5, and C4ˈ. The compound has been found in >70 plant species, including grapes, peanuts, and berries [250,251], as well as in red wine. The mechanism of resveratrol’s biological action is based on numerous cell signaling pathways—it can arrest the cell cycle, inhibit proliferation, adhesion, and metastasis, induce apoptosis, or reduce inflammation. Resveratrol is involved in the inactivation of the PI3K/AKT/ERK1/2 pathway, inhibits cyclin D1 expression, has estrogenic effects (ER*α* agonist), may enhance the effects of cytostatic agents [191,252], and may play a role in protection against POF [253].

Wu et al. [190] verified the potential of resveratrol to stimulate the repair of oogonial stem cell damage caused by cyclophosphamide and busulfan. The authors showed that resveratrol administered at a dose of 30 mg/kg/day could reduce the loss of these cells and attenuate chemotherapy-induced apoptosis in mouse ovaries [190]. The ability of this compound to prevent the activation of PMFs and reduce cyclophosphamide-induced apoptosis was further proven in a study on rats [191]. The same research group also disclosed the promising protective properties of resveratrol on granulosa cells in in vitro tests [209]. Interestingly, resveratrol ameliorated doxorubicin-induced damage to mouse ovaries by increasing the percentage of primary and antral follicles and reducing the percentage of atretic follicles [192].

Other studies have determined the antioxidant potential of resveratrol in protecting the ovaries from cisplatin-induced damage, but the results have been inconclusive. While most researchers have observed in tests performed on rats that resveratrol supplementation provides dose-dependent protection of ovarian function [193,194,195,196], others have not confirmed the polyphenol’s protective properties in this regard [254]. According to Said et al. [194], resveratrol protects ovaries from the toxic effects of cisplatin by preventing the loss of granulosa cells, reducing PARP-1 expression, and regulating inflammatory and apoptotic events associated with the effects of the alkylating cytostatic.

### 3.4. Synthetic Compounds

#### 3.4.1. Bortezomib

Bortezomib (Figure 6) is a 26S proteasome inhibitor used mainly against hematologic malignancies. Given the use of bortezomib as an adjuvant agent in chemotherapy that does not interfere with oncology drugs, it seems interesting to study the potential use of this compound as a prophylactic agent to protect ovarian function.

A study in an animal model by Roti Roti et al. [197] proved that bortezomib could protect the ovaries from the toxic effects of doxorubicin. After exposure to doxorubicin, bortezomib prolonged fertility time in animals and improved the health of pups [197]. Later studies showed that the use of bortezomib could reduce the number of PMFs and the number of antral follicles compared to the control group [255]. Bortezomib increased the expression of caspase 3 and Ki67, while the expression of the hormone receptors ER*α* and PR was downregulated [255]. Bortezomib acted on granulosa layer cells, inducing apoptosis of these cells and reducing the ovarian reserve [255]. Further clarifying studies in this regard, therefore, seem necessary.

#### 3.4.2. Dexrazoxane

**Figure 7:**
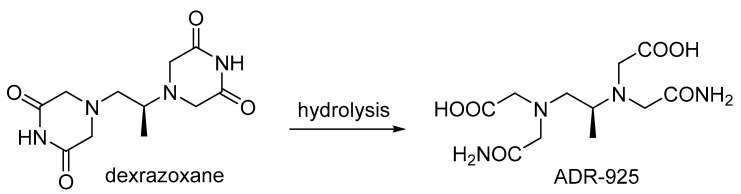
Structure of ADR-925—the hydrolysis product (active metabolite) of dexrazoxane.

Dexrazoxane (Figure 6) is a drug that protects the heart from the cardiotoxic effects of anthracyclines such as doxorubicin [256] thanks to the iron-cation-chelating properties exhibited by its hydrolysis product, ADR-925 (Figure 7). Dexrazoxane does not have the effect of reducing the bioactivity of anthracyclines, while in addition to its cardioprotective effect, it also shows anticancer properties (topoisomerase II inhibitor) [257]. Based on the results of studies in animal models, other beneficial effects of dexrazoxane in combination with cytostatics include protection of the ovaries from DNA damage, prolongation of reproductive viability after chemotherapy, and improved offspring health [258].

Dexrazoxane protected the ovaries from doxorubicin toxicity, improving reproductive health in a mouse model [198]. The use of this drug reduced the extent of DSBs and follicular cell death [198]. Low-dose dexrazoxane not only protected the ovaries but also provided improved mice survival after chemotherapy exposure [198]. In a study on a mouse immortalized cell line derived from the granulosa layer (KK-15), dexrazoxane was shown to prevent H2AFX activation and increase cell viability [210]. On the other hand, Salih et al. [211] set out to verify whether the positive effects of dexrazoxane observed in mouse tests could be translated to non-human primates. To this end, ovarian tissue from a marmoset (a primate) was subjected to in vitro tests, which proved that dexrazoxane could prevent primary doxorubicin-induced DNA damage and subsequent cellular responses to this damage, in addition to attenuating early apoptotic signaling in ovarian cells [211].

#### 3.4.3. Gonadoliberin Analogs

The use of gonadotropin-releasing hormone (GnRH) agonists to protect ovarian function from the toxic effects of chemotherapeutic agents has attracted a great deal of interest from the scientific community. However, the results obtained, including those from clinical trials and meta-analyses of randomized clinical trials, appear inconclusive [259,260,261,262,263,264]. While one study indicates a significant reduction in the incidence of chemotherapy-induced POF after administration of a GnRH agonist [265], another study does not support these conclusions [266]. Possible potential protective mechanisms of action of GnRH agonists include vascular effects (reduced blood flow to and within the ovary) and increased levels of anti-apoptotic molecules [259,267,268,269]. GnRH agonists neutralize the ovotoxic effects of doxorubicin by directly affecting granulosa cells [270], as well as inhibiting follicle pool utilization [271]. According to some authors, the use of GnRH agonists may be effective against hematologic malignancies and breast cancer. However, it would be essential to use GnRH antagonists in combination with GnRH agonists to assess the ovarian reserve before chemotherapy [272].

Goserelin is an example of a synthetic agonist gonadoliberin analog whose potential ovarian-protective properties have been widely described in the scientific literature. In vitro and in vivo tests have indicated the ability of goserelin to inhibit ovarian tumor cell proliferation and simultaneously protect ovarian function from the toxic effects of cisplatin [199]. Although some authors have not observed a protective effect of goserelin against ovarian damage caused by cytostatic drugs in an animal model [273], studies in oncology patients seem promising.

In an analysis by Yuan et al. [274] involving 579 Chinese patients of 20–45 years of age treated with standard chemotherapy, i.e., 4–6 cycles of paclitaxel plus cisplatin, for cervical cancer, the ovarian reserve status (AMH, FSH, E2) was determined, and goserelin administration was included in the study group. Improvements in ovarian reserve function were demonstrated in women using goserelin compared to a control group treated with cytostatics alone [274]. In contrast, Wang et al. [275] evaluated the effect of goserelin on ovarian function in 149 women of 18–45 years of age undergoing chemotherapy for breast cancer (stage I–III). All of the women had regular menstruation and normal ovarian reserve results [275]. However, the 73 patients receiving goserelin (3.6 mg administered subcutaneously every 4 weeks throughout the chemotherapy treatment period) showed better results than the control group of 76 patients [275]. A study after one year showed normal AMH in 46.5% of women in the study group and 21.8% in the control group [275]. Goserelin administration to young women undergoing treatment for early breast cancer may prevent premature menopause [276], protect against ovarian failure [277], and be associated with a lower risk of long-term amenorrhea and a greater likelihood of pregnancy [278,279]. Among women treated with goserelin, a statistically significant increase in the percentage of women menstruating one year after treatment compared to after 2 years of treatment was observed (*p* = 0.006) [280]. A combined use of goserelin and tamoxifen did not have a similar protective effect on the ovarian function [280]. Beneficial effects of goserelin use have also been reported in young women with other types of cancer, such as lymphoma [281].

Kim et al. [282] demonstrated that goserelin’s ability to protect the ovaries during cyclophosphamide- and doxorubicin-based chemotherapy in a group of patients younger than 40 years old with breast cancer was comparable to that demonstrated by leuprorelin (leuprolide acetate). An analysis of clinical trials among Asian breast cancer patients confirmed that leuprorelin could preserve ovarian function, reduce symptoms of ovarian failure, prevent the occurrence of early menopause, and shorten the time to the resumption of menstruation [283]. In a retrospective study, Hoyos-Martinez et al. [284] evaluated the effect of leuprolide on protecting the ovarian reserve in adolescent girls undergoing chemotherapy. The use of leuprolide was associated with higher AMH levels in the group with a lower risk of gonadotoxicity (95% CI 0.97–4.51, *p* = 0.004) [284]. In turn, several other studies have determined the effect of leuprolide on ovarian function among young women battling breast cancer [285,286,287]. Treatment with leuprolide combined with chemotherapy reduced the risk of premature menopause [285], prevented ovarian failure [286], and restored menstruation [287]. While one can find literature reports indicating that leuprolide lacks protective properties against chemotherapy- and radiation-induced ovarian damage [125], they are not numerous and concern in vitro studies.

On the other hand, a combined administration of low doses of triptorelin (3.8 mg/kg), i.e., a GnRH agonist often used as a hormone-responsive anticancer drug, with cyclophosphamide resulted in a significant increase in the number of PMFs and primary, secondary and antral follicles in mice compared to a group of animals receiving the alkylating cytostatic alone (*p* < 0.05) [200]. When using 10-fold-higher doses of triptorelin, the difference was statistically significant for PMFs and primary follicles (*p* < 0.001) [200]. In addition, triptorelin attenuated follicle loss induced by the use of 5-fluorouracil, which was associated with decreased levels of E2, FSH, Bax, and the nuclear factor NF-*κ*B, as well as increased serum levels of AMH and Bcl-2 [201].

One can also find reports on the clinical effects of triptorelin among cancer patients undergoing chemotherapy. Based on the results of a randomized trial on a group of premenopausal women with stage I–III breast cancer, Del Mastro et al. [265] found that inducing temporary ovarian suppression with triptorelin reduced the incidence of chemotherapy-induced early menopause. Lambertini et al. [288] noted that the addition of triptorelin to chemotherapy was associated with a greater likelihood of return of ovarian function in young women with hormone-receptor-positive or hormone-receptor-negative breast cancer, with no statistically significant difference in the incidence of pregnancy. On the other hand, the results of some studies indicate that the rate of amenorrhea with triptorelin was comparable to that observed in the control group (no triptorelin during adjuvant chemotherapy) [289].

#### 3.4.4. Imatinib

Imatinib (Figure 6) is a BCR-Abl tyrosine kinase inhibitor used in the targeted treatment of chronic myeloid leukemia. Abl is considered a sensor of DNA damage, and its activation can lead to the activation of TAp63-*α*, a homolog of p53 expressed in the oocyte [119]. Administration of cisplatin to the ovaries of newborn mice resulted in the accumulation of Abl and TAp63-*α* in the oocyte and its death, while pharmacological inhibition of Abl with imatinib resulted in reduced oocyte death in response to the alkylating cytostatic [119]. Other authors have confirmed that imatinib can protect the ovaries from cisplatin-induced damage in studies on the ovaries of newborn mice [70] or in vitro culture and subrenal grafting of mouse ovaries [212]. On the other hand, the use of imatinib prior to ovarian stimulation increased the oocyte maturity and fertilization rate among mice treated with cyclophosphamide [290]. Hong et al. [213] confirmed that the simultaneous administration of imatinib and cyclophosphamide preserved the ability to produce AMH in the in vitro model studied and had no effect on acquiring metaphase II oocytes.

However, in-depth studies on the potential ovoprotective properties of imatinib appear to be necessary, especially in the light of studies indicating possible ovarian dysfunction during treatment with this cytostatic drug [291,292], as well as those denying the protective effects on PMFs and impaired fertility [293,294].

#### 3.4.5. Metformin

Metformin (Figure 6) is a dimethyl biguanide derivative commonly used in the treatment of type 2 diabetes, especially when accompanied by overweight or obesity. In addition to the antidiabetic effects of metformin, the compound has attracted interest for its extremely promising anti-aging, anti-inflammatory, and anticancer properties [295,296], as well as recently documented properties that may protect ovarian function.

A mouse study showed that the deleterious effects of cyclophosphamide on the ovaries were reduced after the oral administration of metformin [202]. The number of follicles was higher in the group taking cyclophosphamide in combination with metformin than in the group taking the alkylating cytostatic alone (number of PMFs, 16.7 ± 6.3 vs. 9.6 ± 4.7, *p* = 0.004; number of tertiary follicles, 5.4 ± 1.1 vs. 2.6 ± 1.8, *p* = 0.002; corpus luteum, 8.2 ± 1.5 vs. 5.6 ± 1.3, *p* = 0.029) [202]. Serum AMH levels and the number of offspring were also higher in the group taking metformin [202]. A similar beneficial effect of metformin on reducing the ovotoxic effects of cyclophosphamide use was also confirmed in another study [203]. Also in a mouse model, Yang et al. [204] showed that intragastric administration of metformin could alleviate ovarian damage and endocrine disruption caused by chemotherapeutic agents, with these effects being due to metformin’s reduction of oxidative stress and inflammation-related damage. On the other hand, Ayhan et al. [205], in a study on rats, showed that metformin, presumably through its antioxidant effects, could mitigate ovarian damage caused by carboplatin.

#### 3.4.6. Tamoxifen

Tamoxifen (Figure 6) is a selective, non-steroidal estrogen receptor modulator that has found use in molecularly targeted therapy for breast cancer [297]. In a rat study, tamoxifen administration reduced follicle loss caused by cyclophosphamide and doxorubicin [206]. Similarly, beneficial effects of tamoxifen have been also reported in studies on ovarian cultures of newborn rats [214]. However, the molecular mechanisms underlying the ovoprotective effects of tamoxifen during chemotherapy remain unclear.

Less promising effects of tamoxifen have been observed in cancer patients. Admittedly, the compound has not been found to significantly affect the oocyte count [298] or ovarian reserve in women with breast cancer [299,300], but according to Shandley et al. [299], tamoxifen use may affect the chances of having a child in this group of patients.

## 4. Conclusions

Women of reproductive age undergoing chemotherapy are at risk of irreversible ovarian failure. Current methods of determining the ovarian reserve do not accurately predict the future reproductive potential of patients undergoing chemotherapy. Among the most ovotoxic cytostatics are alkylating agents, mainly cyclophosphamide, but the adverse effects of drugs belonging to other groups have also been widely described in the scientific literature. Experimental studies, however, should focus not only on PMFs but also on follicles at other stages of maturation, which are present in the ovaries at any time and are affected by chemotherapy. Moreover, it is difficult to determine the gynotoxic effects of individual components in the case of multidrug therapies.

The degree of ovarian damage may depend on several factors, including age, the treatment regimen, and the doses of cancer drugs used. Therefore, doctors must know the effects of chemotherapeutics planned for use on women’s future fertility before starting treatment. Anticancer drugs can damage the female reproductive system by affecting the ovarian follicles or stroma. Since the exact mechanisms of ovarian damage are still unclear, further studies are needed to clarify them unequivocally.

Given the increasing number of women affected by POF as a result of chemotherapy, fertility preservation for patients after cancer treatment is of considerable importance. Standard fertility preservation methods include the cryopreservation of embryos, unfertilized oocytes, or ovarian tissue. However, these methods do not guarantee the maintenance of ovarian function in the long term. In this context, pharmacological protection of the ovaries from the toxic effects of drugs with selected protective agents administered before or during chemotherapy could be an excellent solution.

The potential agents that protect ovarian function include selected hormones, agents that affect the activity of apoptotic pathways and modulate gene expression, and several selected natural and synthetic compounds. Hormones and GnRH agonists are of particular interest to researchers because they can act selectively without interfering with physiological mechanisms or the efficacy of chemotherapy. However, the results on the protective properties of selected agents come mainly from in vitro studies or those on animal models (mice, rats). By searching the ClinicalTrials.gov database, we found two studies investigating the effects of goserelin or tamoxifen on ovarian function in women with breast cancer (NCT02430103 or NCT01384526, respectively), but the results have not been published yet. Thus, large-scale clinical trials to prove the efficacy of these agents among women of reproductive age still need to be conducted. There is also a need for studies aimed at standardization in terms of the models used, follicle classification, dose size, time and route of administration, number of time points, and repetitions. Although these differences make it difficult to draw firm conclusions from the studies, it is reasonable to undertake further extensive work on the possible use of selected agents in protecting ovarian function during gynotoxic chemotherapy.

## Figures and Tables

**Table 1:** Gynotoxic properties of selected anticancer drugs in studies performed on animal models.

Drug	Model	Dose	Treatment Duration	Gynotoxic Effects	Ref.
Cyclophosphamide	3-week-old ICR mice	100 mg/kg (i.p.)	Single dose	Decrease in the number of follicles, impaired hemostasis of oocyte quality, reduced ability to develop an embryo	[35]
	3-week-old B6C3F1 mice	120 mg/kg (i.p.)	Single dose	Deterioration of oocytes, reduction and fibrosis of the ovaries	[36]
	Sprague–Dawley rats	20 mg/kg (i.p.)	Once per day for 10 days	Apoptosis of ovarian cells (apoptosis index [AI] = 13.6%)	[37]
	Pregnant Charles Foster rats (average age: 120 days)	2 mg/kg (i.p.)	Single dose	Prevention of folliculogenesis in offspring resulting in anovulation and infertility	[38]
	Pregnant 129 or L1 mice	7.5 mg/kg (i.p.)	10.5 and 11.5 days of pregnancy	Loss of PMFs and increased follicle growth activation in offspring	[39]
	Swiss albino mice	200 mg/kg (i.p.)	Single dose on the 14th, 21st, or 28th day after birth	Long-term effects on oocyte developmental competence in early but not adult life	[40]
	4–8-week-old CD-1 mice	100 mg/kg (i.p.)	Single dose	Possible negative effects on the fertility of subsequent generations	[41]
	6–8-week-old C57BL/6 mice	70 mg/kg (i.p.)	Single dose	POF induction	[42]
	C57BL/6 mice	300 mg/kg (i.p.)	Single dose	PMF depletion	[43]
	8-week-old C57BL/6 mice	100 mg/kg (i.p.)	6 doses over 2 weeks	Loss of follicles, irreversible deterioration of oocytes	[44]
	Experimental xenografts of human ovaries	75 mg/kg (i.v.)	Single dose	Apoptotic follicle death	[45]
	Experimental xenografts of human ovaries	200 mg/kg (i.p.)	Single dose	Reduction in PMFs	[46]
Cisplatin	6-week-old CD-1 mice	2 mg/kg (i.p.)	Once per day for 15 days	Reduction in the total number of follicles in the ovaries, especially PMFs	[47]
	8-week-old C57BL/6 mice	2.5 mg/kg (i.p.)	Once per day for 5 days → one week break → once per day for 5 days	Increased number of atretic follicles, decreased number of antral follicles	[48]
	PN10 and PN50 of C57BL/6J mice	2 or 4 mg/kg (i.p.)	Single dose	Mitochondrial dysfunction in oocytes	[49]
	C57BL/6 mice	5 mg/kg (i.p.)	Single dose	PMF depletion	[43]
	Mature Sprague-Dawley mice	0.5, 1, 1.5, 2, 3 or 4 mg/kg (i.p.)	Once a day for 10 days	Disorders of the estrous cycle, lack of mature follicles	[50]
	5–6-week-old rats	5 mg/kg (i.p.)	Single dose	Reduction in PMFs	[51]
5-Fluorouracil	6–8-week-old C57BL/6 mice	150 mg/kg (i.p.)	Single dose	Mild ovotoxic effects	[52]
	26–30-day-old C57BL/6J mice	450 mg/kg (i.p.)	Single dose	Decreased survival of preantral follicles	[53]
	8–9-week-old C57BL/6J mice	125 mg/kg (i.p.)	3-fold injection	Progressive atresia of growing follicles, reduction in ovarian volume, no change in number of PMFs	[54]
	8-week-old ICR mice	50 mg/kg (i.p.)	Once per day for 4 days	Inhibition of oocyte maturation and early embryonic development	[55]
Doxorubicin	5-day, 21-day or 8-week CD-1 mice	10 mg/kg (i.p.)	Single dose	Ovarian reserve depletion, PMFs atresia, excessive PMF activation	[56]
	7–8-week-old ICR mice	10 mg/kg (i.v.)	Single dose	Effects on oocytes	[57]
	4-week-old or 7–8-week-old ICR mice	7.5 or 10 mg/kg (i.p.)	Single dose	Reduction in the size and weight of the ovary, reduction in ovulation, reduction in the population of PMFs and secondary follicles	[58]
	6-week-old or 10–22-week-old C57BL/6 or 129/Sv mice	10 mg/kg (i.p.)	Single dose	Impaired expression of collagen IV, less differentiated luteal cells (smaller cytoplasm, reduced expression of StAR)	[59]
	Sprague-Dawley rats	15 mg/kg (cumulative dose; i.p.)	7 injections	Decrease in the number of follicles, decrease in the volume of the ovaries and the uterus	[60]
Irinotecan	2-month-old ICR mice	100 mg/kg (i.p.)	Single dose	Mild toxicity to the ovaries	[61]
	8-week-old MCH mice	20, 100 or 500 µg/mouse (i.p.)	Single dose	Increased apoptotic processes in ovarian follicles	[62]
Paclitaxel	6–8-week-old Wistar rats	5 mg/kg (i.p.)	5 doses at 3-day intervals	Reduction in the number of antral follicles	[63]
	7-week-old ICR mice	30 mg/kg (i.p.)	Single dose	Destruction of antral follicles on day 1 after chemotherapy	[64]
	2-month-old Wistar rats	4.6 mg/kg (MTD, i.v.)	Single dose	Destruction of PMFs and follicles bilaterally and multilaterally, decreased ovarian reserve, increased fetal mortality	[65]
	5–6-week-old rats	7.5 mg/kg (i.p.)	Single dose	Reduction in the number of PMFs	[51]
Docetaxel	8-week-old CD-1 mice	5 or 10 mg/kg (i.p.)	2 doses (1st and 14th day)	Reduction in ovarian weight, number of secondary follicles, and total number of follicles	[66]
	6–8 week B6 mice	30 mg/kg (i.p.)	Single dose	Reduction in total number of follicles, destruction of ovarian structure	[67]

**Table 2:** Gynotoxic properties of selected anticancer drugs in studies performed in vitro, including in human ovarian tissues.

Drug	Model	Dose	Treatment Duration	Gynotoxic Effects	Ref.
Cyclophosphamide	Metaphase II mouse oocytes	10.25 µM	45-min incubation	Mitochondrial membrane damage in oocytes	[68]
	Human ovary cortex sections taken from premenopausal cancer patients	0.5–500 µg/mL	2–48-h incubation	Damage to granular cell nuclei, changes in basement membrane (depending on cyclophosphamide concentration)	[34]
Cisplatin	Follicles from ovaries of 13-day-old C57BL/6 mice	10^−2^, 10^−3^, or 10^−4^ µM	13-day incubation	Decrease in survival and growth of follicles	[69]
	Ovaries of newborn mice	0.1, 0.5, 1, or 5 µg/mL	24-h incubation	Ovarian damage, loss of follicles	[70]
	Ovaries of young CD-1 mice	0.5, 1, or 5 μg/mL	24-h incubation	Reduction in number and condition of follicles, damage to PMFs and granulosa cells	[71]
	Human granulosa cells (COV434, HGrC1, HLGC)	20, 40, or 100 µg/mL	140-h incubation	Induction of apoptosis in mitotic non-luteinized and non-mitotic luteinized granulosa cells	[72]
	Biopsies of human ovaries	5 or 10 μg/mL	6-day incubation	Deterioration of follicle health, increased cell apoptosis, reduced proliferation	[73]
5-Fluorouracil	Isolated preantral mouse ovarian follicles	0.3, 1, 3, 10, or 30 mM	Maximum 12-day incubation	Negative effects on follicle growth, estradiol production, and oocyte maturation	[74]
Doxorubicin	Granulosa cells from ovaries of 21–23-day-old ICR mice	0.4, 0.8, or 1.6 μg/mL	24-h incubation	Induced cell apoptosis, increase in ROS levels, decrease in mitochondrial membrane potential	[75]
	Follicles from ovaries of 7–8-week-old ICR mice	10 µM	2-h incubation	Effects on oocytes	[57]
	Multilayered secondary ovarian follicles of CD-1 mice	2, 20, 100, or 200 nM	24-h incubation	Dose-dependent toxicity for follicle growth and survival, as well as 17*β*-estradiol secretion	[76]
	Multilayered secondary ovarian follicles of rats	200 nM	Single dose	Ca^2+^ release from the endoplasmic reticulum through activation of Src kinase	[77]
	Human primary granulosa cells	0.01, 0.05, 0.1, 0.2, or 0.5 μg/mL	48-h incubation	Induction of cytotoxicity and miR-132 expression in granulosa cells	[11]
	Biopsies of human ovaries	1 or 2 μg/mL	6-day incubation	Deterioration of follicle health, increased cell apoptosis, reduced proliferation	[73]
Paclitaxel	Early secondary follicles taken from BDF1 mice	2.5 × 10^−4^, 2.5 × 10^−3^, or 2.5 × 10^−2^ µM	12-day incubation	Reduced growth of preantral or more mature follicles (depending on paclitaxel concentration)	[78]
	Follicles from ovaries of 13-day-old C57BL/6 mice	10^−8^–10^−10^ M	13-day incubation	Reduced survival and growth of ovarian follicles	[69]
Docetaxel	Neonatal ovaries taken from C57Bl/6J mice	0.1, 1, or 10 µM	24-h incubation	Negative effects on early growing follicles, reduction of PMFs, induction of somatic cell apoptosis	[79]

**Table 3:** Potential protective mechanisms against the ovotoxic effects of selected chemotherapeutics in studies performed in animal models.

Protective Factor	Drug	Model	Protective Mechanisms	Ref.
Anti-Müllerian hormone	Cyclophosphamide	6-week-old Swiss mice	Protection against loss of PMFs	[168]
	Cyclophosphamide	6-week-old mice	Reducing follicle activation, protecting follicle reserve, improving long-term fertility and reproductive outcomes	[169]
	Cyclophosphamide	8-week-old C57/B6 mice	Protection of ovarian reserve	[170]
	Cyclophosphamide, carboplatin, doxorubicin	6–7-week-old nu/nu mice	Protection of ovarian reserve by blocking activation of PMFs	[6]
	Cyclophosphamide	Heterotransplantation of human ovarian tissue from a 12-year-old female donor	Protection of ovarian reserve	[170]
Ghrelin	Cisplatin	Wistar rats	Preservation of the number of PMFs and primary follicles	[171]
	Cisplatin	5-week-old ICR mice	Synergistic protective effects of ghrelin and melatonin against follicular damage and ovarian failure	[172]
Luteinizing hormone (lutropin)	Cisplatin	4- or 5-week-old mice	Protecting PMFs reserve, preserving fertility at reproductive age	[173]
Melatonin	Cisplatin	7-week-old C57BL/6J mice	Mitigating ovarian damage	[174]
	Cisplatin	8-week-old C57BL/6 mice	Reducing toxicity to ovaries, preserving long-term fertility	[175]
	Cisplatin	3-month-old Wistar rats	Modulating ovarian dysfunction, preserving fertility	[176]
	Cyclophosphamide	3- or 6-week-old ICR mice	Prevention of ovarian granulosa cell loss	[177]
	Cyclophosphamide	8-week-old Swiss mice	Prevention of PMFs loss, reduction of cell apoptosis and oxidative damage in the ovary	[178]
	Cyclophosphamide	Sprague-Dawley rats	Protecting ovaries from damage by activating the Hippo pathway	[179]
Sphingolipids (C1P, S1P)	Cyclophosphamide	6–8-week-old mice	Reduction of ovarian damage	[102]
	Busulfan	8-week-old FVB/NJNarl mice	Protection of PMFs, partial preservation of ovarian function	[180]
	Dacarbazine	6–8-week-old BALB/c mice	Preserving the number of PMFs	[181]
	Cyclophosphamide, doxorubicin	Heterotransplantation of human ovarian tissue fragments	Protection of follicles from chemotherapeutic-induced apoptosis	[45]
	Cyclophosphamide	Human fetal ovarian xenografts	Prevention of follicle apoptosis, maintenance of the PMF population in transplants	[122]
MicroRNA	Cyclophosphamide, busulfan	6-week-old ICR mice	Prevention of follicular atresia	[182]
Quercetin	Cyclophosphamide	5–6-week-old C57BL/6 mice	Reducing follicle loss and apoptosis of growing follicles	[183]
	Cyclophosphamide	6–8-week-old C57BL/6 mice	Protection of ovarian reserve by reversing dysfunction and activating mitochondrial biogenesis, regulating pyroptosis	[184]
	Cyclophosphamide	12-week-old Sprague-Dawley rats	Restoring ovarian function, inhibiting oxidative stress	[185]
	Cyclophosphamide	10–12-week-old Wistar rats	Protection of early-stage and total follicles	[186]
	Cisplatin	Wistar rats	Ovarian protection through antioxidant, anti-inflammatory, and anti-apoptotic activities	[187]
Rapamycin (sirolimus)	Cyclophosphamide	8-week-old BALB/c mice	Prevention of follicle activation	[109]
	Cyclophosphamide	5-week-old C57BL/6 mice	Protection of PMFs, reduction of follicular cell apoptosis	[188]
	Cisplatin	4-week-old BALB/c mice	Protection of PMFs	[189]
Resveratrol	Cyclophosphamide, busulfan	6-week-old C57BL/6 mice	Prevention of oogonial stem cell loss, reduction of ovarian cell apoptosis	[190]
	Cyclophosphamide	7-week-old Sprague-Dawley rats	Prevention of PMF activation, reduction of chemotherapeutic-induced cell apoptosis	[191]
	Doxorubicin	6–8-week-old C57BL/6 mice	Increase in number of primary and antral follicles, decrease in number of atretic follicles, preservation of number of PMFs	[192]
	Cisplatin	Sprague-Dawley rats	Protection of ovarian follicles, increasing levels of progesterone, folliculotropic hormone, and LH	[193]
	Cisplatin	3-week-old Sprague-Dawley rats	Mitigation of follicle loss and AMH lowering, inhibition of inflammatory mediator elevation	[194]
	Cisplatin	12–14-week-old Wistar rats	Mitigation of oxidative stress, inflammation, and cell apoptosis	[195]
	Cisplatin	Wistar rats	Maintaining the number of PMFs and primary follicles	[196]
Bortezomib	Doxorubicin	4-week-old CD-1 mice	Preventing accumulation of chemotherapeutics in ovaries, reducing DNA damage, prolonging fertility time	[197]
Dexrazoxane	Doxorubicin	4-week-old CD-1 mice	Protection of ovaries, improvement of reproductive health	[198]
Goserelin	Cisplatin	6-week-old BALB/c nu/nu mice	Shortening of estrous cycles, prolongation of estrous duration, protection of PMF and preantral follicles	[199]
Triptorelin	Cyclophosphamide	10-week-old mice	Increase in the number of PMFs, primary, secondary and antral follicles	[200]
	5-Fluorouracil	50-day-old Sprague-Dawley rats	Protection against ovarian damage by modulating hormones, Bax, Bcl-2, and NF-*κ*B	[201]
Imatinib	Cisplatin	5-day CD1 mice	Oocyte protection	[119]
Metformin	Cyclophosphamide	6–8-week-old C57BL/6 mice	Preservation of ovarian function and fertility in mice	[202]
	Cyclophosphamide	6–8-week-old C57BL/6 mice	Increase in number of antral follicles, AMH levels, and litter size	[203]
	Cyclophosphamide together with busulfan	6-week-old mice	Protection against ovarian damage, reducing oxidative damage and inflammation	[204]
	Carboplatin	3-month-old Wistar rats	Increased AMH levels and number of PMFs	[205]
Tamoxifen	Cyclophosphamide, doxorubicin	4–6-week-old Sprague-Dawley rats	Reduction in follicle loss, improvement in reproductive function	[206]

**Table 4:** Potential protective mechanisms against the ovotoxic effects of selected chemotherapeutics in studies performed in vitro.

Protective Factor	Drug	Model	Protective Mechanisms	Ref.
Anti-Müllerian hormone	Cyclophosphamide	Human ovarian cortex biopsies	Reduction in follicle damage (by about half) and PI3K activation	[207]
MicroRNA	Cyclophosphamide (active metabolite)	Mouse ovaries	Reduction of ovarian cell apoptosis	[208]
Rapamycin (sirolimus)	Cisplatin	Rat ovaries	Inhibition of activation and protection of PMFs	[189]
Resveratrol	Cyclophosphamide (active metabolite)	Primary cultures of rat granulosa cells	Reduction of oxidative stress levels	[209]
Dexrazoxane	Doxorubicin	Mouse cell line KK-15	Protection against DNA damage, increase in cell viability	[210]
	Doxorubicin	Marmoset ovarian tissue	Protection from DSBs, increase in number of granulosa cells in antral follicles	[211]
Imatinib	Cisplatin	Ovaries of newborn mice	Protection of ovarian follicles	[70]
	Cisplatin	In vitro culture and subrenal grafting of mouse ovaries	Oocyte protection	[212]
	Cyclophosphamide	Preantral mouse follicles	Protection of AMH production, no effect on metaphase II oocyte retrieval	[213]
Tamoxifen	Cyclophosphamide	Ovaries of newborn rats	Counteracting follicle loss	[214]
